# Supplementary figures and images for: The clinical value of carcinoembryonic antigen for tumor metastasis assessment in lung cancer
Source: PeerJ. 2019 Aug 7;7:e7433. doi: 10.7717/peerj.7433 (PMC6689222; doi:10.7717/peerj.7433)

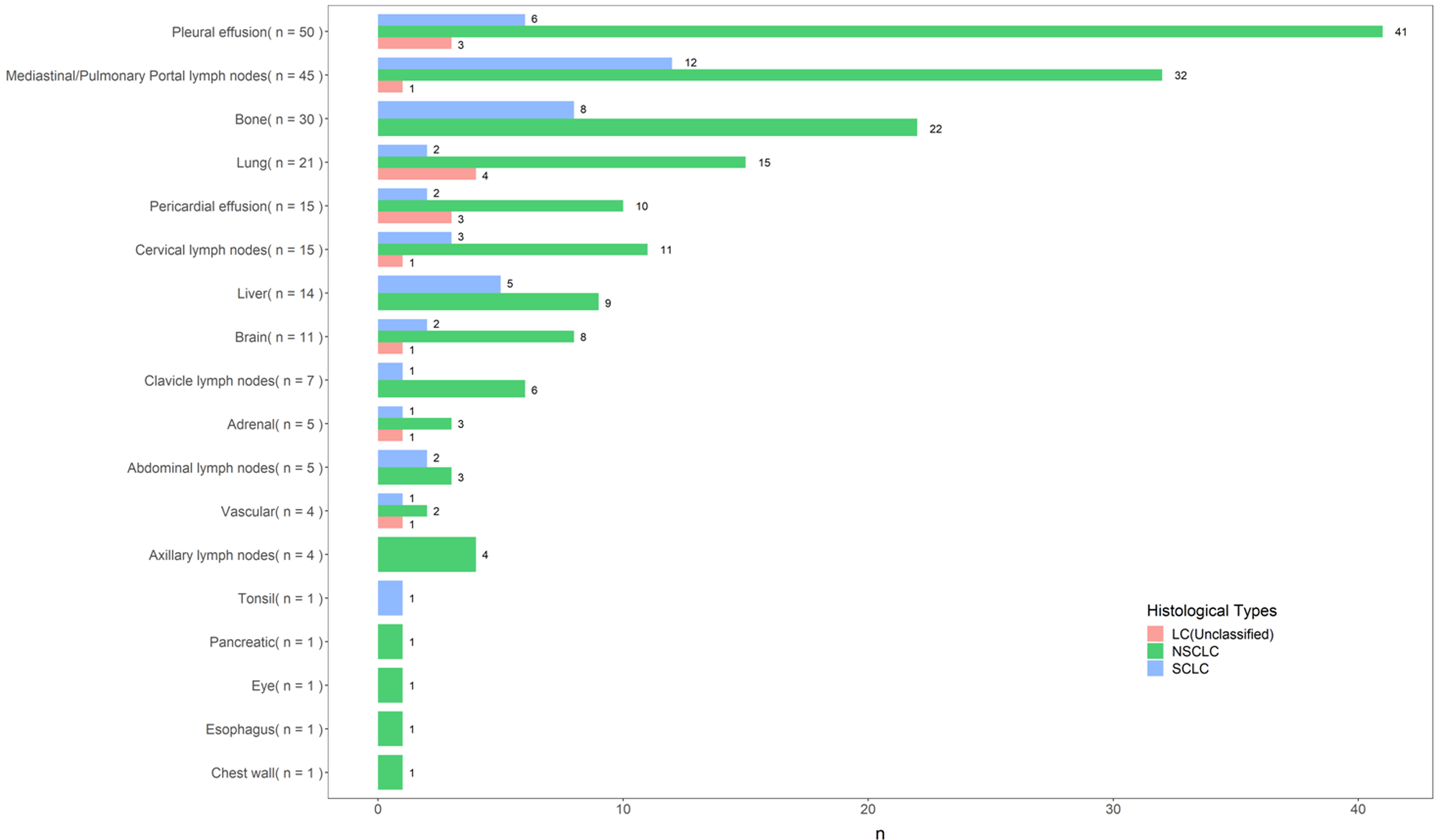

Supplement: Figure S1 [file peerj-07-7433-s002.png]

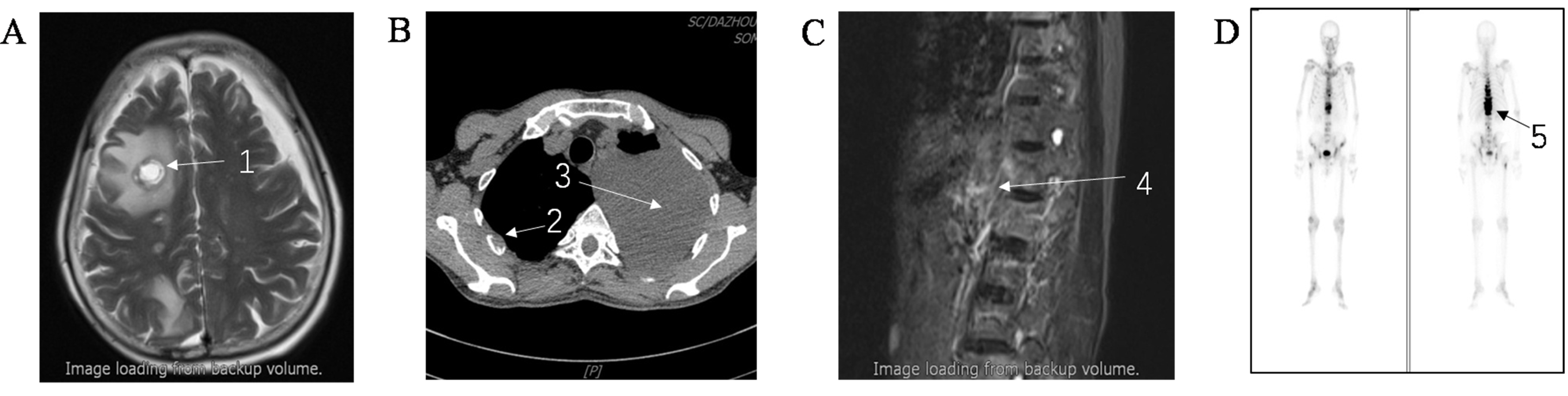

Supplement: Figure S2 — (A) 1: Brain metastasis. (B) 2: Rib metastasis, 3: Massive pleural effusion. (C) 4: Pyramidal bone metastasis. (D) 5: Bone metastasis. [file peerj-07-7433-s003.png]

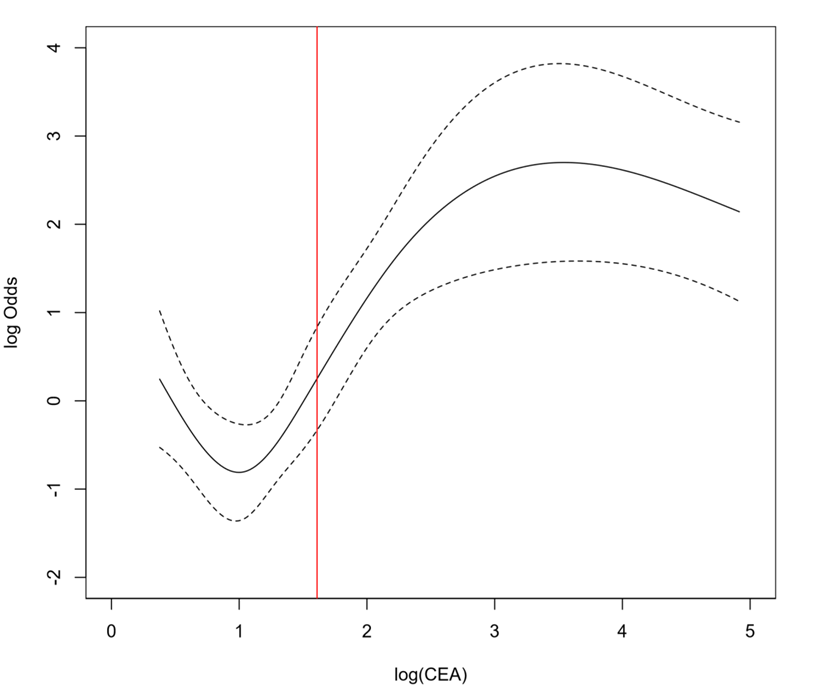

Supplement: Figure S3 [file peerj-07-7433-s004.png]

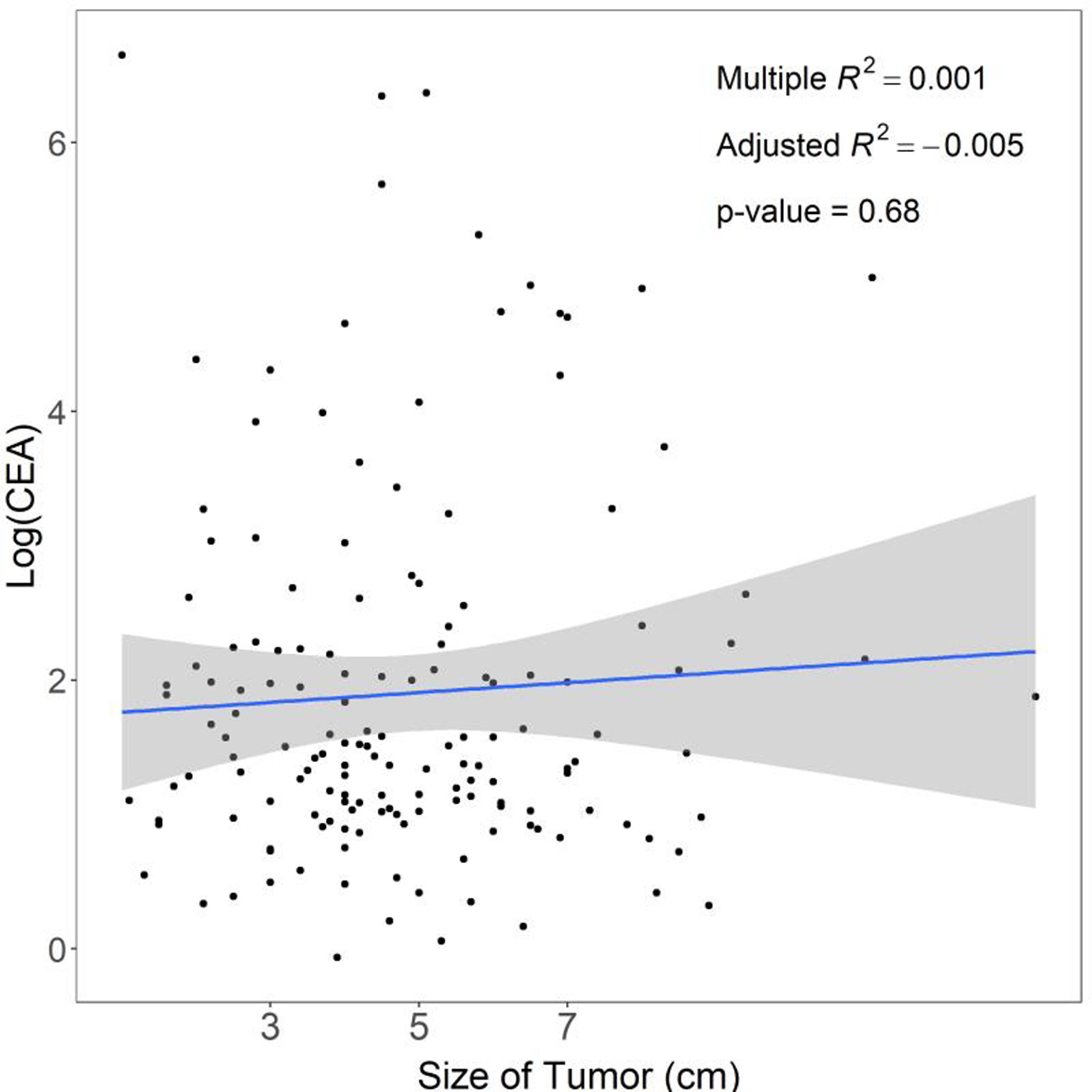

Supplement: Figure S5 [file peerj-07-7433-s006.png]
